# Quantitative Evaluation of Tendon Gliding Sounds and Their Classification Using Deep Learning Models

**DOI:** 10.7759/cureus.81790

**Published:** 2025-04-06

**Authors:** Daiji Nakabayashi, Atsuyuki Inui, Yutaka Mifune, Kohei Yamaura, Tatsuo Kato, Takahiro Furukawa, Shinya Hayashi, Tomoyuki Matsumoto, Takehiko Matsushita, Ryosuke Kuroda

**Affiliations:** 1 Department of Orthopaedic Surgery, Kobe University Graduate School of Medicine, Kobe, JPN

**Keywords:** artificial intelligence, deep learning, digital stethoscopes, machine learning, tendon gliding sounds

## Abstract

This study aims to develop and evaluate a deep learning (DL) model for classifying tendon gliding sounds recorded using digital stethoscopes (Nexteto, ShareMedical, Japan, Nagoya). Specifically, we investigate whether differences in tendon excursion and biomechanics produce distinct acoustic signatures that can be identified through spectrogram analysis and machine learning (ML). Tendon disorders often present characteristic tactile and acoustic features, such as clicking or resistance during movement. In recent years, artificial intelligence (AI) and ML have achieved significant success in medical diagnostics, particularly through pattern recognition in medical imaging. Leveraging these advancements, we recorded tendon gliding sounds from the thumb and index finger in healthy volunteers and transformed these recordings into spectrograms for analysis. Although the sample size was small, we performed classification based on the frequency characteristics of the spectrograms using DL models, achieving high classification accuracy. These findings indicate that AI-based models can accurately distinguish between different tendon sounds and strongly suggest their potential as a non-invasive diagnostic tool for musculoskeletal disorders. This approach could offer a non-invasive diagnostic tool for detecting tendon disorders such as tenosynovitis or carpal tunnel syndrome, potentially aiding early diagnosis and treatment planning.

## Introduction

Biomechanical sounds produced by the human body during movement have long been a focus of clinical research [1,2]. Muscle contractions generate mechanical vibrations detectable as muscle sounds (mechanomyograms) [1,2]. In tendon disorders, palpation often reveals tactile sensations like clicking or resistance, suggesting that tendon gliding also produces characteristic sounds. While muscle sound assessment is well-documented, tendon gliding sounds remain largely unexplored.

With the rapid progress of artificial intelligence (AI) and machine learning (ML), these technologies are increasingly applied in medical diagnostics, particularly image analysis. Shinohara et al. demonstrated deep learning (DL) potential in diagnosing carpal and cubital tunnel syndromes with high accuracy using ultrasound images [3,4]. Ashinsky et al. used MRI to predict early symptomatic knee osteoarthritis [5], while Xue et al. explored DL's diagnostic value in hip osteoarthritis [6]. Zhang et al. assessed a DL approach for detecting anterior cruciate ligament lesions via arthroscopy [7]. Kalmet et al. reviewed DL applications in bone fracture detection and classification [8], and Tomita et al. investigated its use for automatic detection of osteoporotic vertebral fractures in CT scans [9].

In oncology, Jabeen et al. employed DL to classify breast cancer via ultrasound imaging [10]. Tsai et al. demonstrated AI’s effectiveness in BI-RADS classification for screening mammography [11], while Zhang et al. highlighted its role in detecting lymph node metastases in breast cancer patients using convolutional neural networks [12]. These advancements underscore AI and ML’s transformative potential in enhancing diagnostic precision across various medical fields, from musculoskeletal disorders to oncology.

 In particular, DL models have proven effective in identifying patterns and diagnosing conditions such as carpal tunnel syndrome (CTS) through ultrasound imaging [3,4]​. Given this progress, AI-driven approaches could provide a non-invasive means of diagnosing tendon disorders by analyzing tendon gliding sounds. Specifically, we hypothesized that the anatomical differences between the thumb (flexor pollicis longus, FPL) and the index finger (flexor digitorum superficialis/profundus, FDS/FDP) in terms of tendon excursion and gliding distance would result in distinct tendon gliding sounds. The purpose of this study is to analyze and classify tendon gliding sounds using DL and explore its potential application in non-invasive musculoskeletal diagnostics.

## Materials and methods

Participants

Based on a power analysis performed using G*Power software (Version 3.1.9.7; Heinrich Heine University Düsseldorf, Germany) with an assumed effect size of 0.5, α=0.05, and power=0.8, the required sample size was estimated to be 12 participants (24 hands). Accordingly, 12 healthy volunteers (nine male volunteers and three female volunteers) with no history of tendon or musculoskeletal disorders were recruited for this study. The mean age was 36.5±2.86 years (range: 34-45 years). 

Data collection

**Figure 1:**
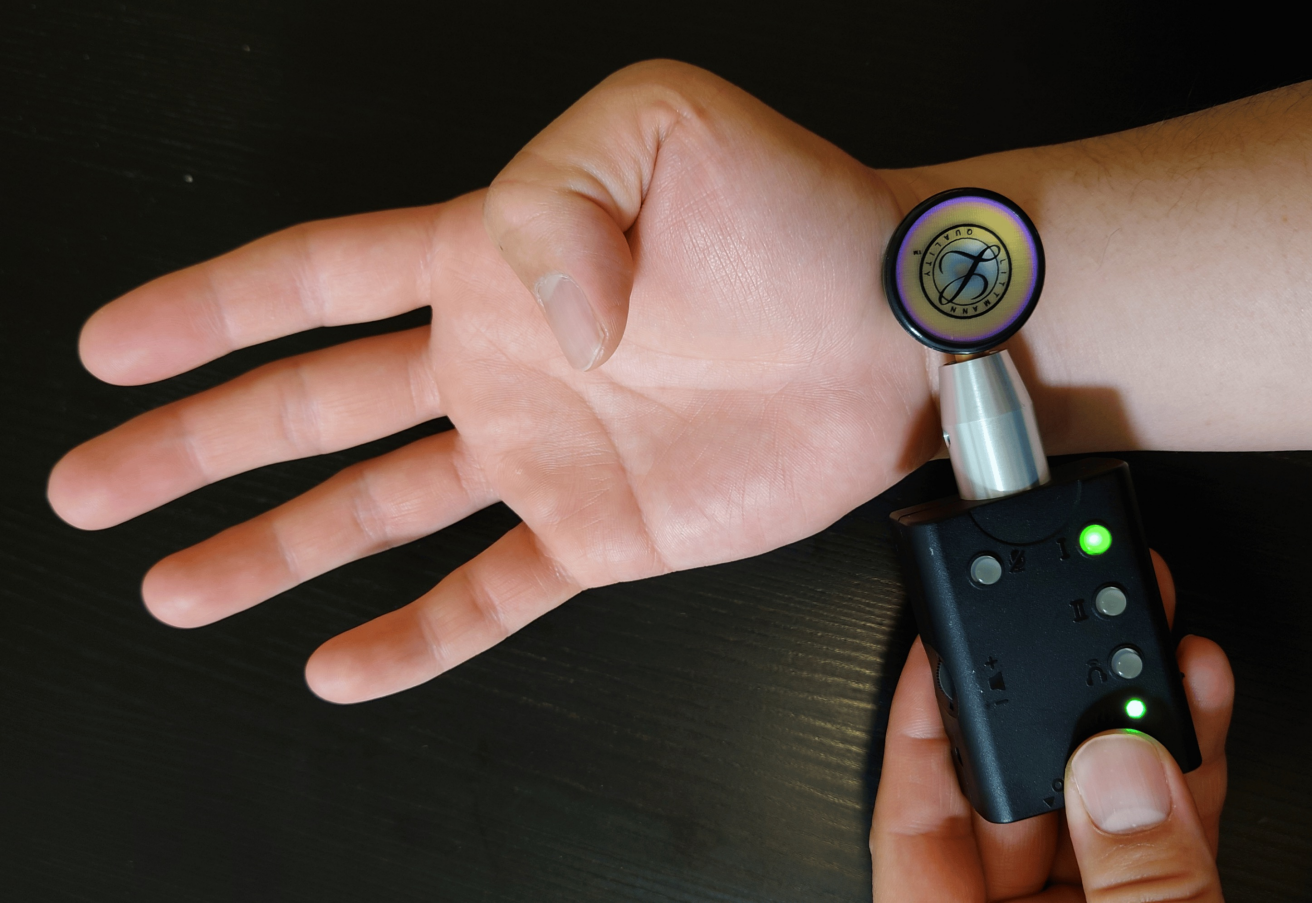
Location of tendon sound recording using a digital stethoscope.

Participants performed automatic flexion-extension movements of the thumb interphalangeal (IP) joint and index proximal interphalangeal (PIP) joint in sync with a metronome set at a rhythm of 60 beats per minute (BPM) (Figure 1). Each movement sequence was repeated 10 times for each finger. Tendon gliding sounds were recorded using a digital stethoscope (Nexteto, ShareMedical, Japan, Nagoya) placed at the intersection of the wrist crease and radial flexor carpi (Figure 1). All recordings were conducted in a soundproof room, and the same experienced orthopedic surgeon managed both the recordings and synchronization with the metronome.

**Figure 2:**
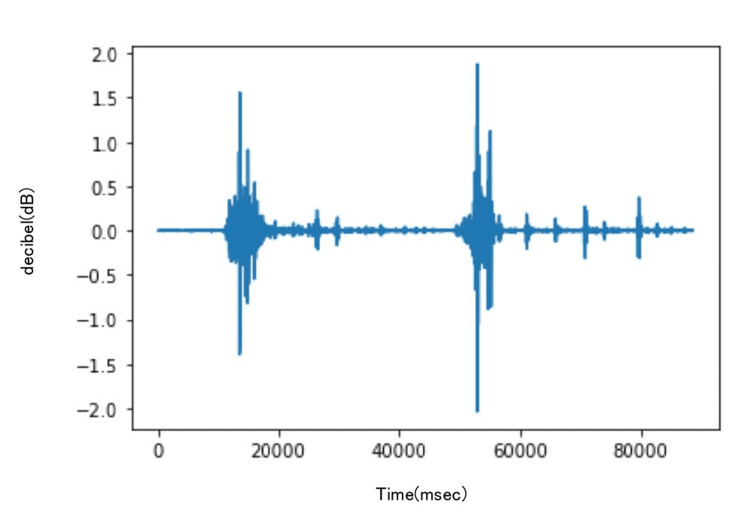
Tendon gliding sound recordings of the thumb using Audacity. Audacity: Audacity Team, Pittsburgh, USA

**Figure 3:**
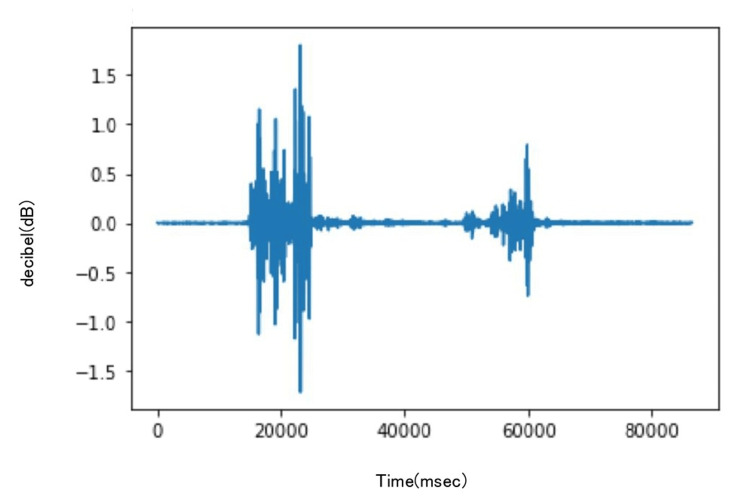
Tendon gliding sound recordings of the index finger using Audacity. Audacity: Audacity Team, Pittsburgh, USA

The recordings were made using Audacity (Audacity Team, Pittsburgh, USA) (Figures 2, 3), and the sound data were processed using the Python library SciPy for Fourier transformation. The sounds were filtered using a band-pass filter ranging from 10 Hz to 2048 Hz to eliminate friction noise between the skin and the stethoscope.

**Figure 4:**
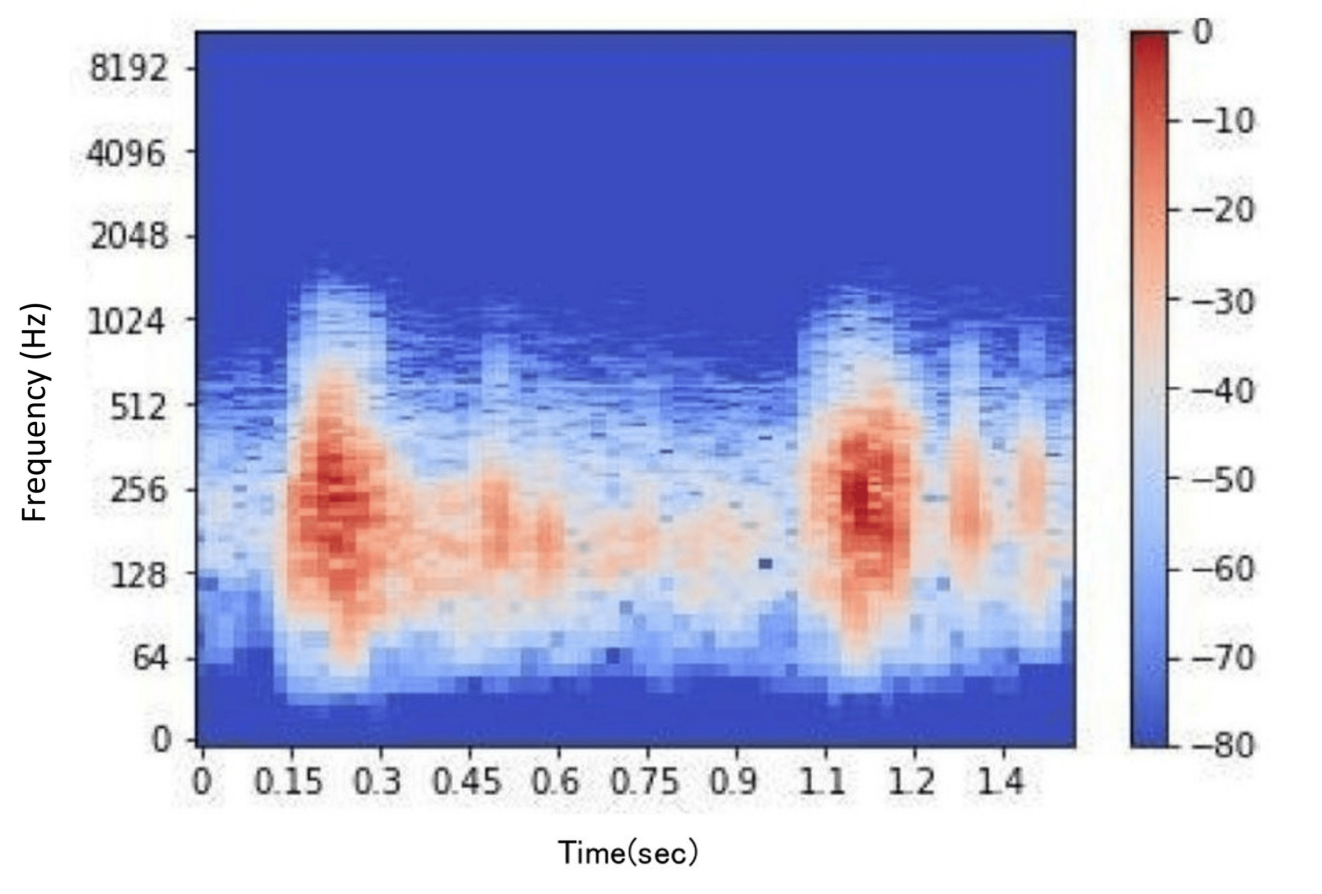
Spectrogram of tendon gliding sounds for the thumb.

**Figure 5:**
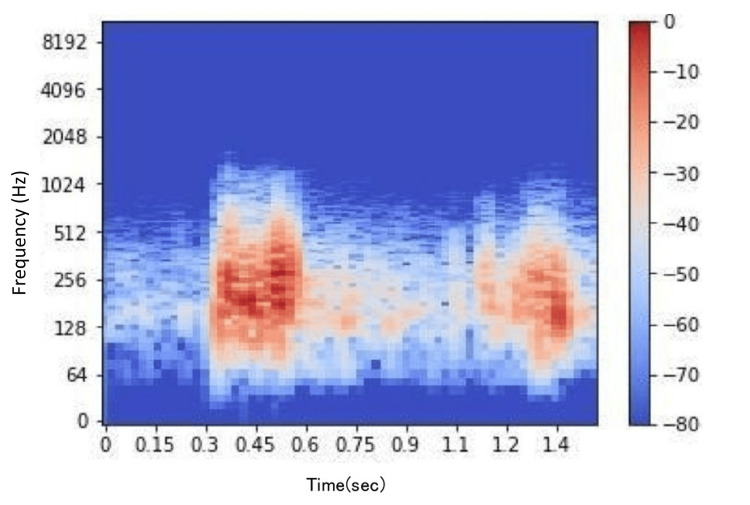
Spectrogram of tendon gliding sounds for the index finger.

Spectrograms were generated from the sound data using a short-time Fourier transform (STFT) implemented with SciPy (Figures 4, 5). Although detailed data and code are not publicly available due to privacy and patent considerations, the program was developed entirely using basic functions from SciPy. In this study, tendon gliding sounds were recorded using a digital stethoscope, and the audio data were processed using Fourier transform. This allowed us to extract the frequency components of the tendon gliding sounds and compare and analyze the differences between the thumb and index finger. Conventional Fourier transform has the drawback of losing time information; however, STFT performs Fourier transforms over short time intervals, enabling visualization of how frequencies change over time. This approach allowed for both numerical and visual analyses of the characteristics of tendon gliding sounds.

**Figure 6:**
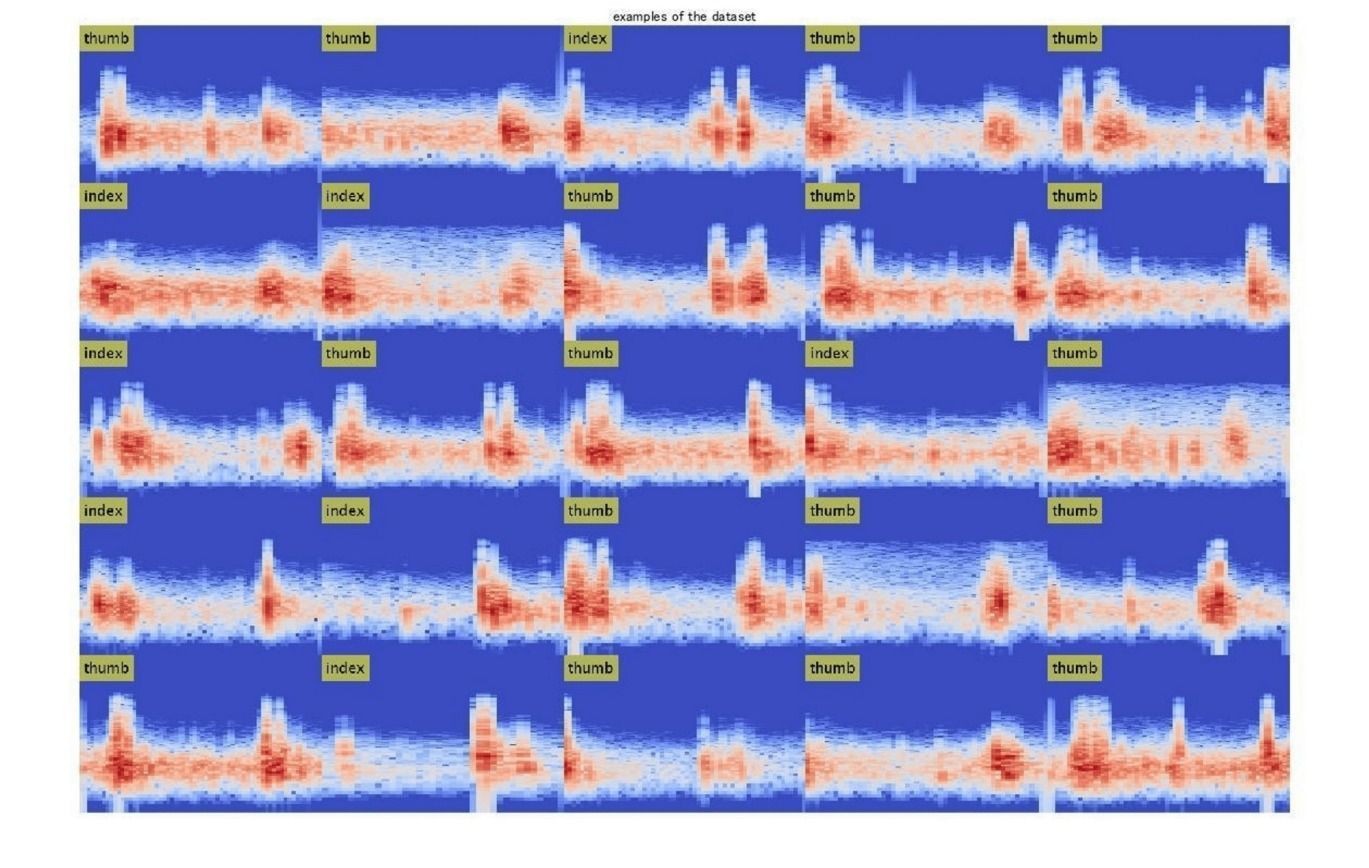
The dataset displays spectrograms of tendon gliding sounds collected from the thumb and index finger during flexion-extension movements.

Following this, the obtained sound data were analyzed using the Python audio processing library Librosa (ver. 0.10.2). Metrics such as average frequency, central frequency, spectral centroid, spectral flatness, and zero-crossing rate were calculated. These spectrograms and metrics formed the dataset for subsequent DL model training and evaluation (Figure 6).

DL model

Transfer learning was performed using three pre-trained models: ResNet101, MobileNet_v2, and EfficientNet. These models are widely used for medical imaging data and have been optimized to reduce computation time and memory usage. The number of convolutional layers in each model is 53 for MobileNet_v2, 99 for ResNet101, and 82 for EfficientNet. Confusion matrices were obtained for each model using the training dataset. The image features that the DL models focused on were visualized as heatmaps and overlaid on the original images. In this study, occlusion sensitivity was employed to visualize the important features detected by the network.

Training and validation

The dataset was divided into training (70%), validation (15%), and test (15%) sets. Stratified sampling ensured balanced representation of thumb and index finger data. Five-fold cross-validation was used during the training phase to further enhance the robustness of the model.

Measures

The accuracy of the three learning models was evaluated using test data. The evaluation terms were defined as follows: accuracy (percentage of correct answers for all data), precision (percentage of the patient group correctly judged), recall (percentage of data correctly judged as the patient group, same as sensitivity), specificity (percentage of data correctly judged as the control group), and F-measure (the harmonic mean of accuracy and recall).

## Results

Acoustic analysis

Frequency analysis revealed clear and statistically significant differences in the acoustic characteristics of tendon gliding sounds between the thumb and index finger (Table 1). These results indicate that the thumb produces lower-frequency sounds compared to the index finger, supporting the hypothesis that tendon sounds vary by type and can be quantified.

**Table 1:** Frequency analysis results for the thumb and index finger.

Parameter	Thumb	Index	p-value
Average frequency (Hz)	263.7± 55.8	272.0 ± 69.2	0.07
Central frequency (Hz)	236.5 ± 57.7	252.0 ± 71.5	0.004
Spectral centroid	-13.8 ± 1.3	-14.0 ± 0.2	0.045x10⁻⁵
Spectral flatness	-14.0 ± 0.6	-13.3 ± 1.7	0.003
Zero-crossing rate	0.02± 0.0	0.01 ± 0.0	0.004

Average Frequency

Average frequency indicates the overall average pitch of the tendon gliding sounds. The thumb showed a slightly lower average frequency (263.7 ± 55.8 Hz) compared to the index finger (272.0 ± 69.2 Hz), although this difference was not statistically significant (p = 0.07).

Central Frequency

Central frequency represents the center point of the frequency spectrum, reflecting the central value of the sound's energy distribution. The thumb exhibited a significantly lower central frequency (236.5 ± 57.7 Hz) compared to the index finger (252.0 ± 71.5 Hz; p = 0.004).

Spectral Centroid

Spectral centroid represents the center of mass of the frequency spectrum, reflecting the proportion of high-frequency components. The thumb had a more negative spectral centroid value (-13.8 ± 1.3) than the index finger (-14.0 ± 0.2), indicating a shift toward lower frequencies. This difference was highly significant (p = 0.045 × 10⁻⁵).

Spectral Flatness

Spectral flatness evaluates the tonal quality of the signal. Higher values indicate more noise-like components, while lower values indicate a more tonal signal. The thumb exhibited lower spectral flatness (-14.0 ± 0.6) compared to the index finger (-13.3 ± 1.7), suggesting a smoother sound profile (p = 0.003).

Zero-Crossing Rate

Zero-crossing rate measures the number of times the acoustic signal crosses zero along the time axis, reflecting the speed of frequency changes. The thumb showed a lower zero-crossing rate (0.02 ± 0.0) compared to the index finger (0.01 ± 0.0), reflecting slower frequency variations (p = 0.004). 

DL classification

**Figure 7:**
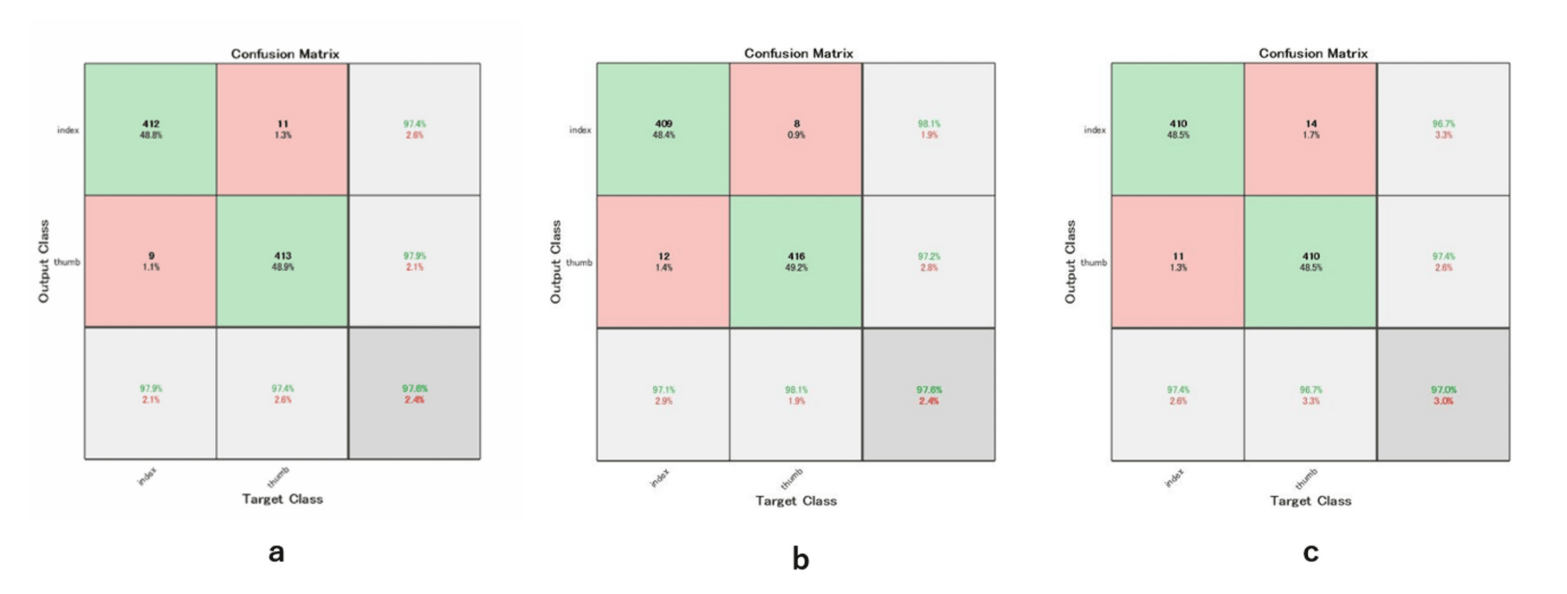
The confusion matrices (a: MobileNet, b: ResNet101, c: EfficientNet). A confusion matrix is a performance evaluation tool that visualizes the classification results by comparing predicted labels with actual labels. It indicates the number of correctly and incorrectly classified cases for each class, allowing detailed assessment of model performance beyond overall accuracy.

**Figure 8:**
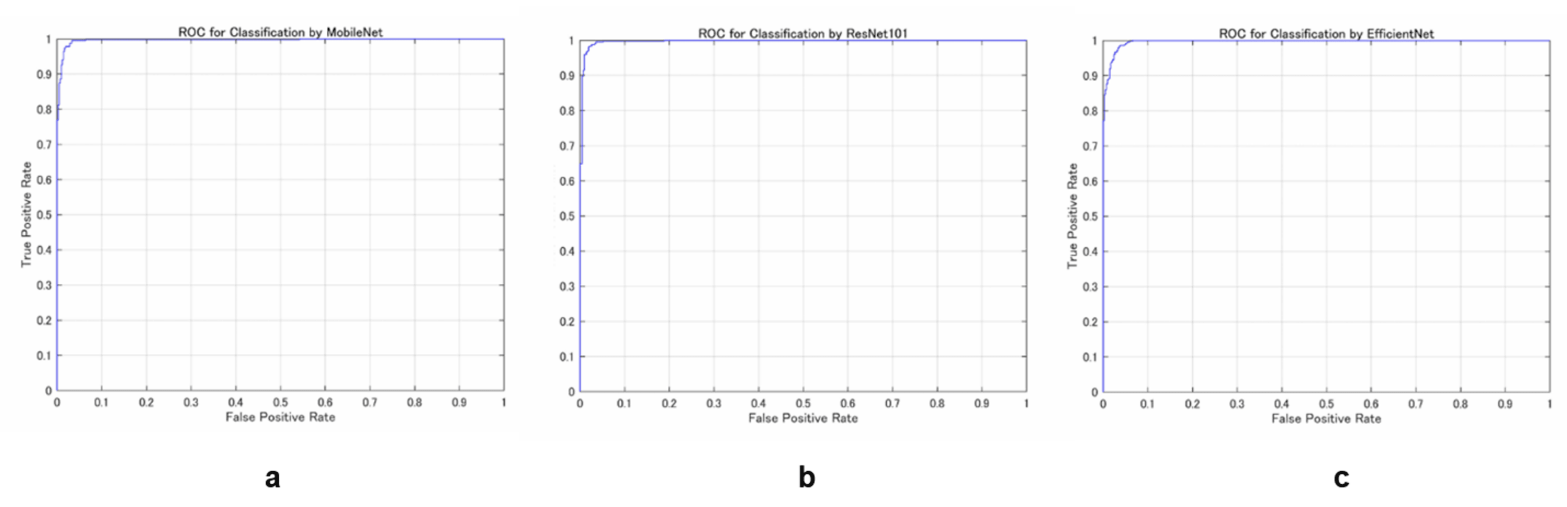
The receiver operating characteristic (ROC) curves (a: MobileNet, b: ResNet101, and c: EfficienNet). The receiver operating characteristic (ROC) curve illustrates the relationship between the true positive rate and false positive rate, providing a visual evaluation of the model’s classification performance. An AUC (area under the curve) value closer to 1 indicates higher classification accuracy.

The classification performance of the three DL models was comprehensively evaluated using confusion matrices and receiver operating characteristic (ROC) curves (Figures 7, 8).

A summary of the predictive metrics for each model is provided in Table 2.

**Table 2:** Performance metrics of the MobileNet_v2, ResNet101, and EfficientNet models.

Metric	MobileNet_v2	ResNet101	EfficientNet
Accuracy	0.9763	0.9763	0.9704
Recall	0.9787	0.9720	0.9739
Precision	0.9741	0.9811	0.9670
F-measure	0.9764	0.9765	0.9704
AUC (ROC)	0.9964	0.9969	0.9967

In the classification of tendon gliding sounds, MobileNet_v2 achieved an accuracy of 0.9763, with a precision of 0.9741 and a recall of 0.9787. The area under the ROC curve (AUC) was 0.9964. ResNet101 demonstrated comparable performance, with an accuracy of 0.9763, a slightly higher precision of 0.9811, and an AUC of 0.9969. EfficientNet yielded marginally lower classification performance, with an accuracy of 0.9704, a precision of 0.9670, and an AUC of 0.9967.

**Figure 9:**
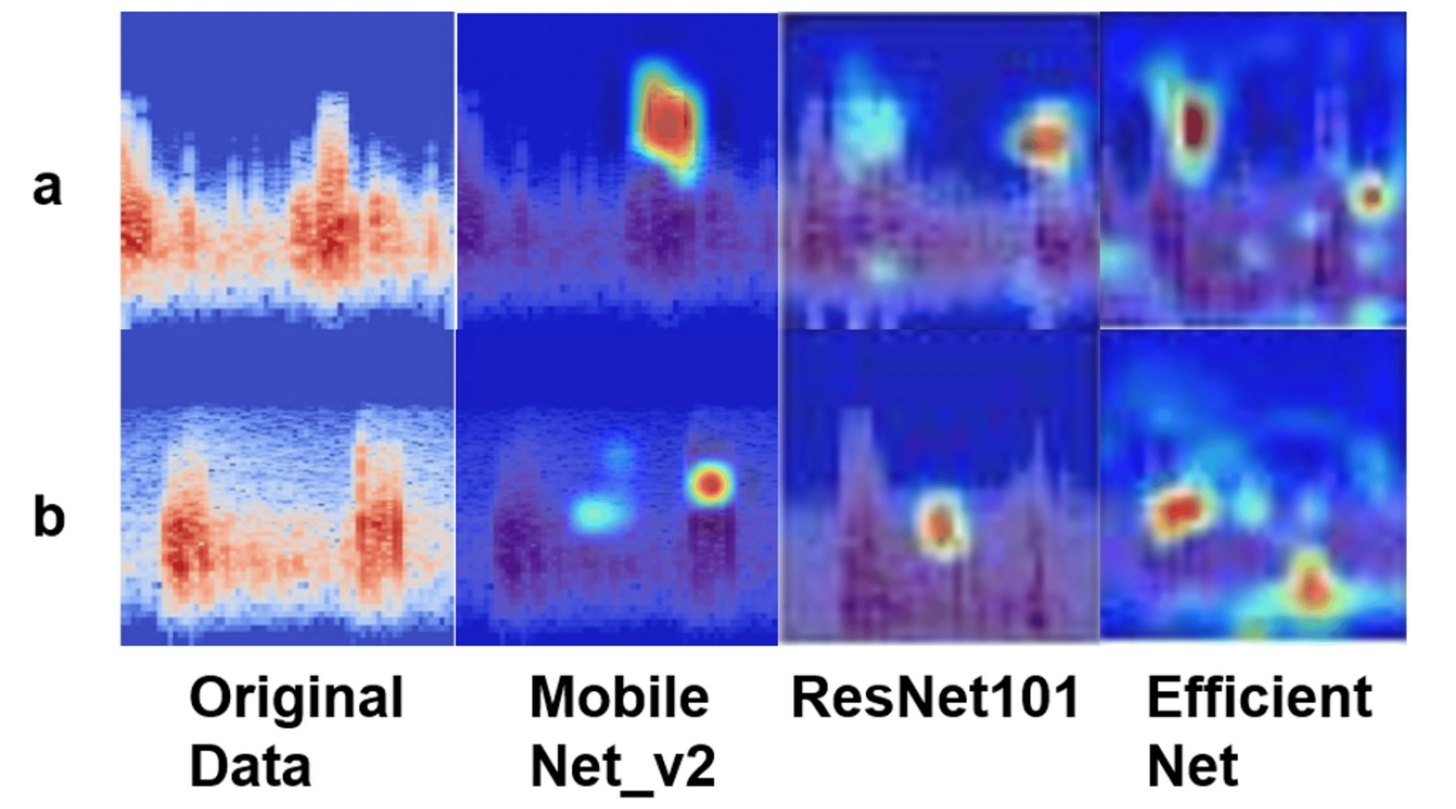
Comparison of original spectrogram and occlusion sensitivity analysis results. The figure shows the original spectrograms of sound data recorded during flexion and extension movements, along with the results of occlusion sensitivity analysis performed by MobileNet_v2, ResNet101, and EfficientNet. The thumb is labeled as A, and the index finger as B. The occlusion sensitivity analysis confirmed that each model focused on the high-frequency bands during thumb flexion and extension movements.

Confusion matrix analysis revealed low misclassification rates across all models, with MobileNet_v2 exhibiting the least interclass confusion between thumb and index finger categories. No statistically significant differences in AUC values were observed among the three models. Although it remains challenging to quantitatively identify specific frequency bands contributing to classification, visualization via occlusion sensitivity provided qualitative insight into the regions emphasized by the models. Heatmaps overlaid on the spectrograms indicated that the models primarily focused on high-frequency components when predicting the presence or absence of sounds during thumb flexion and extension (Figure 9).

## Discussion

The objective of this study was to investigate whether differences in the acoustic characteristics of tendon gliding sounds can be identified and classified using DL models, thereby exploring their potential application in non-invasive musculoskeletal diagnostics. This study is based on the hypothesis that “anatomical and functional differences in tendons are reflected in their acoustic properties, which can be learned and classified by DL models.”

Acoustic analysis revealed clear differences in the frequency distribution between the thumb and index finger. All classification models, MobileNet_v2, ResNet101, and EfficientNet, demonstrated high classification performance. Although ResNet101 showed a slightly higher precision (0.9811), the difference in the F1-score between ResNet101 (0.9765) and MobileNet_v2 (0.9764) was minimal. Notably, MobileNet_v2 achieved these results with significantly less training time and computational resources, indicating its suitability for use in resource-constrained clinical settings. ROC curves for all models indicated high sensitivity and precision, and MobileNet_v2 showed minimal loss fluctuation during training, resulting in a smoother and more stable learning curve. These results suggest that MobileNet_v2 is a practical and robust model for classifying tendon gliding sounds and support its implementation in portable or embedded diagnostic devices.

Recent studies have demonstrated that DL can detect subtle patterns in musculoskeletal sounds and images that are difficult for humans to discern, thereby enhancing diagnostic accuracy [3,4]. The observed differences in frequency characteristics between the thumb (FPL) and index finger (FDS/FDP) are likely due to anatomical and biomechanical variations in tendon displacement and gliding distance [13-15]. The FPL operates the thumb with a relatively short gliding distance, while the FDS/FDP control the index finger with a longer gliding range. Such differences in the gliding distance significantly affect the force generation and mechanical efficiency of the muscle-tendon unit. Brand et al. have stated that muscles with longer tendons and gliding distances can transmit force over a broader range of motion, exhibiting unique mechanical characteristics [14]. Furthermore, the thumb's role in fine grasping and its higher dependency on the FPL may contribute to the lower frequency characteristics of its acoustic spectrum, possibly reflecting differences in mechanical load and stress distribution compared to the index finger [16].

Considering the structure of the surrounding tendon sheath, the relationship between tendon anatomy and sound generation becomes clearer. In studies of tendon gliding, the FPL of the thumb is encapsulated in a more complex pulley system, which reduces friction while enabling acoustically distinct gliding [14,15]. These biomechanical insights align with the present findings, in which the gliding sound of the thumb exhibited lower spectral frequencies compared to the index finger. Frequency analysis further revealed significant differences in parameters such as center frequency, spectral centroid, and zero-crossing rate, indicating that anatomical and functional differences in tendons distinctly influence their acoustic properties.

Additionally, results from occlusion sensitivity analysis contributed to the interpretability of the classification processes of each DL model. By masking specific regions of the spectrogram and analyzing changes in classification accuracy, critical frequency bands for distinguishing between thumb and index finger sounds were identified. This helped visualize the previously “black-box” classification process and highlighted key acoustic features relevant to tendon gliding sound identification [17]. Though quantitative evaluation remains challenging, this visualization particularly emphasized the importance of high-frequency bands in thumb movement and confirmed that each model focused on relevant acoustic regions.

These model interpretations of acoustic feature differences reflect anatomical and biomechanical characteristics of tendons and strongly suggest the potential for clinical diagnostic applications. Non-invasive techniques employing digital stethoscopes and ML algorithms may allow for differentiation of normal and pathological conditions through acoustic patterns. Future applications and validation are expected in disorders affecting tendon gliding, such as tenosynovitis and carpal tunnel syndrome.

The application of AI and ML in medical diagnostics is rapidly advancing, especially in the fields of image analysis and clinical outcome prediction [18,19]. Recent studies have shown that DL models can accurately diagnose conditions such as CTS from ultrasound images [3,4]. This study demonstrated the feasibility of recording and analyzing tendon gliding sounds using a digital stethoscope. Significant differences in frequency characteristics were found between the thumb and index finger, confirming that each tendon has distinct acoustic features.

Moreover, using AI to classify these sounds has shown that ML models can aid in the diagnosis of musculoskeletal disorders. The high classification performance achieved by each DL model used in this study validates the effectiveness of DL in processing acoustic data [20,21]. As AI becomes more integrated into clinical practice, the development of a novel, non-invasive diagnostic tool for tendon disorders through acoustic analysis is expected. Future steps include conducting clinical trials with a larger number of participants and collaborating with other orthopedic surgeons to analyze and validate tendon gliding sound features not only in healthy individuals but also in pathological states such as tenosynovitis and CTS. Additionally, the goal is to implement this approach in real-time analysis and portable devices to evaluate its utility as a screening tool in clinical settings.

Limitations

This study is a preliminary investigation aimed at clinical application, and to the best of our knowledge, no previous research has specifically focused on tendon gliding sounds. Although the subjects were healthy individuals, the data obtained strongly suggest the potential for clinical application, making this study a groundbreaking attempt. However, several limitations exist.

First, although a power analysis was conducted using G*Power software to determine the required sample size, the dataset used in this study was small, and there is a risk of overfitting in each learning model.

Second, sex-based differences in tendon sounds were not examined. Anatomical characteristics such as tendon and muscle mass, as well as subcutaneous fat, may vary between sexes and potentially influence tendon gliding sounds. Future studies should investigate sex-related acoustic differences to develop more accurate classification algorithms.

Third, although all recordings were performed in a soundproof room, environmental noise may not have been entirely eliminated. Subtle external sounds or device operation noise may have affected the acoustic analysis. For clinical application, it will be necessary to develop noise-resistant data processing methods and robust learning models.

Finally, although the stethoscope was consistently placed at the intersection of the wrist crease and the flexor carpi radialis tendon, the effect of participants’ body types (e.g., obese or lean) on acoustic characteristics was not considered. Differences in subcutaneous fat and muscle thickness may affect sound transmission, and this should be addressed in future research.

Despite these limitations, the findings of this study demonstrate the feasibility of non-invasive analysis and classification of tendon gliding sounds, laying the groundwork for future validation and application research.

## Conclusions

This study successfully applied digital stethoscopes and DL models to classify tendon gliding sounds, achieving a high accuracy. Significant differences in acoustic characteristics between the thumb and index finger were identified, demonstrating the feasibility of non-invasive diagnostics for musculoskeletal disorders. These findings highlight the potential of AI in clinical orthopedics for precise and innovative diagnostic tools. Future research is expected to expand on the use of AI to analyze, for example, the acoustic characteristics of tendon gliding sounds between healthy subjects and patients with CTS, and whether non-invasive diagnosis using tendon gliding sounds is feasible.

## Appendices

This study was designed as a pilot investigation to explore the feasibility of using digital stethoscopes and deep learning models for tendon gliding sound analysis. The primary goal was to establish a methodological framework and assess the potential of AI-based classification before conducting a larger-scale study.
